# Random forest predictive modeling of prolonged hospital length of stay in elderly hip fracture patients

**DOI:** 10.3389/fmed.2024.1362153

**Published:** 2024-05-17

**Authors:** Hao Liu, Fei Xing, Jiabao Jiang, Zhao Chen, Zhou Xiang, Xin Duan

**Affiliations:** ^1^Department of Orthopedic Surgery, Orthopedic Research Institute, West China Hospital, Sichuan University, Chengdu, China; ^2^Department of Pediatric Surgery, West China Hospital, Sichuan University, Chengdu, China; ^3^Department of Orthopedics Surgery, West China Sanya Hospital, Sichuan University, Sanya, China; ^4^Department of Orthopedic Surgery, The Fifth People’s Hospital of Sichuan Province, Chengdu, China

**Keywords:** machine learning, random forest, hip fracture, prolonged hospital length of stay, prediction mode, elderly

## Abstract

**Background:**

In elderly individuals suffering from hip fractures, a prolonged hospital length of stay (PLOS) not only heightens the probability of patient complications but also amplifies mortality risks. Yet, most elderly hip fracture patients present compromised baseline health conditions. Additionally, PLOS leads to increased expenses for patient treatment and care, while also diminishing hospital turnover rates. This, in turn, jeopardizes the prompt allocation of beds for urgent cases.

**Methods:**

A retrospective study was carried out from October 2021 to November 2023 on 360 elderly hip fracture patients who underwent surgical treatment at West China Hospital. The 75th percentile of the total patient cohort’s hospital stay duration, which was 12 days, was used to define prolonged hospital length of stay (PLOS). The cohort was divided into training and testing datasets with a 70:30 split. A predictive model was developed using the random forest algorithm, and its performance was validated and compared with the Lasso regression model.

**Results:**

Out of 360 patients, 103 (28.61%) experienced PLOS. A Random Forest classification model was developed using the training dataset, identifying 10 essential variables. The Random Forest model achieved perfect performance in the training set, with an area under the curve (AUC), balanced accuracy, Kappa value, and F1 score of 1.000. In the testing set, the model’s performance was assessed with an AUC of 0.846, balanced accuracy of 0.7294, Kappa value of 0.4325, and F1 score of 0.6061.

**Conclusion:**

This study aims to develop a prognostic model for predicting delayed discharge in elderly patients with hip fractures, thereby improving the accuracy of predicting PLOS in this population. By utilizing machine learning models, clinicians can optimize the allocation of medical resources and devise effective rehabilitation strategies for geriatric hip fracture patients. Additionally, this method can potentially improve hospital bed turnover rates, providing latent benefits for the healthcare system.

## 1 Background

In recent years, the global aging population has resulted in a steady increase in the number of elderly individuals experiencing hip fractures. These fractures present significant risks to the elderly and are regarded as the most serious type of fracture (1). The rising occurrence of hip fractures is frequently associated with higher mortality and disability rates in the elderly, severely affecting their quality of life and carrying substantial healthcare and societal consequences (2, 3). The risk of hip fractures increases with age, largely due to the rising prevalence of osteoporosis among the elderly, which leads to fragile bones. Osteoporosis, characterized by decreased bone density and strength, significantly increases the likelihood of hip fractures in older adults (4). In those with osteoporosis, fragile bones are prone to breaking from low-energy trauma, unlike the high-energy impacts typically required to cause fractures in younger individuals. Hip fractures in the elderly result in reduced mobility and an elevated mortality rate. In addition to osteoporosis, various factors contribute to the heightened risk of hip fractures in the elderly, such as decreased physical function, polypharmacy, visual impairment, poor coordination and balance, slower reaction times, reduced muscle strength around the hip joint, and ligamentous laxity (5, 6).

The delayed discharge of elderly patients with hip fractures has significant impacts on both the patients and healthcare institutions. Prolonged hospital stays impose substantial burdens on the physical and mental well-being of elderly patients and may strain their economic resources. Shortening hospital stays can reduce early mortality rates (7). Studies show that hospital stays longer than 14 days significantly increase the risk of postoperative mortality within 30 days for elderly patients undergoing hip surgery (7). Additionally, PLOS challenges the healthcare system’s ability to deliver comprehensive and integrated care. Elderly patients with hip fractures have high healthcare demands and require support for early mobilization. Extended hospital stays also increase the risk of various inpatient complications, adding strain to healthcare and nursing services (8). In trauma medicine centers, hospital beds are a vital resource for providing timely and effective care to emergency patients. The constant demand for available beds remains a challenge for healthcare institutions. Beyond delivering high-quality and timely healthcare, medical facilities face the challenge of reducing PLOS and increasing bed turnover rates (BTR) to optimize patient flow and resource utilization (9). While extending hospitalization may initially seem beneficial in reducing early mortality rates, research by Schneider et al. (7) found that PLOS actually significantly increased the risk of mortality within 30 days after surgery for elderly hip fracture patients. Various factors associated with delayed discharge of elderly hip fracture patients have been studied, including delayed surgical timing, gender, cerebrovascular diseases, smoking status, arthritis, postoperative analgesia, ASA classification, age, cognitive decline, multiple fractures, depression, and non-clinical factors (10, 11). Variations in surgical interventions and care occur across different regions, patient populations, and hospitals, leading to differing definitions of PLOS. In our institution’s context, we define PLOS as the 75th percentile of the entire patient cohort in this study, which is 12 days.

The aim of this study is to perform feature selection to identify key variables for predicting delayed discharge in elderly hip fracture patients and to develop a predictive model for PLOS. We will employ the random forest algorithm for model construction and validation, comparing its performance with conventional logistic regression models. The random forest prediction model has significant potential to improve clinical workflows, optimize resource allocation, and enhance medical care for prioritized patients. Healthcare professionals can use the model to tailor personalized rehabilitation plans for high-risk patients, involving interdisciplinary teams to provide comprehensive and timely medical care. This approach can help reduce hospital stays, decrease susceptibility to complications, and improve the efficient use of healthcare resources.

## 2 Materials and methods

### 2.1 Population

This study adopts a retrospective observational cohort design, with the study population comprising individuals whose data were collected from the West China Hospital’s Electronic Health Record (HIS) system between November 2021 and December 2023. Inclusion criteria are defined as follows: (1) inclusion of hip patients aged 65 years and above, (2) patients undergoing surgical treatment during their hospitalization, and (3) patients undergoing surgical procedures involving intramedullary nail fixation or artificial joint replacement. Exclusion criteria are as follows: (1) old fractures, (2) pathological fractures, (3) rapid discharge due to non-clinical factors, (4) transferred patients who have undergone standardized treatment, (5) in-hospital deaths, (6) patients opting for discharge to receive end-of-life care, and (7) patients transferring to other departments for continued treatment. Based on these criteria, a total of 360 patients were included and then randomized into a training set (70% of the cohort) and a testing set (30%).

This study collected factors influencing delayed discharge, primarily encompassing four domains: demographic variables, fracture-related variables, surgery-related variables, medical history variables, and laboratory examination variables. Demographic variables include gender, age, and BMI. Fracture-related variables encompass time from injury to admission, high-energy trauma status, preoperative immobilization, multiple traumas (hip fracture with fractures in other areas), and fracture type. Surgery-related variables cover surgery type, preoperative waiting time, surgery duration, intraoperative bleeding, and transfusion status. Medical history variables list smoking history, alcohol consumption history, osteoporosis, lower limb vascular diseases (venous thrombosis and vascular wall sclerosis), diabetes, hypertension, Alzheimer’s or dementia, heart failure, cardiovascular diseases, chronic obstructive pulmonary disease or pulmonary fibrosis, cerebrovascular diseases, Parkinson’s disease, malignancy, paralysis, and arrhythmias. The variables related to medical history in this study all refer to the patients’ preoperative medical history. Laboratory examination variables were utilized for diagnostic purposes or collection through the assessment of laboratory indicators, encompassing an array of parameters such as anemia, hypoalbuminemia, renal insufficiency, electrolyte imbalances, hepatic dysfunction, hyperlipidemia, total protein (TP), platelets (PLT), white blood cells (WBC), and D-dimer. Laboratory blood test results acquired throughout the patient’s hospitalization were consistently leveraged for diagnosing physiological imbalances and associated disorders. Then, through univariate and multivariate analyses, a preliminary screening identified variables such as anemia, hypoalbuminemia, renal insufficiency, electrolyte imbalances, hepatic dysfunction, and hyperlipidemia. The specific laboratory values were derived by averaging the laboratory blood test results obtained at the time of admission and on the first postoperative day, initially identifying indicators such as TP, PLT, WBC, and D-dimer. This study comprehensively considered the significant impact of surgery on the physical condition of elderly patients, along with the need for timely interventions based on predictions of PLOS post-surgery. Therefore, we have incorporated the results of blood tests conducted on the first day after surgery for the patients. Orthopedic practitioners use physical examinations in conjunction with medical imaging to diagnose hip fractures, further delineating their classification and formulating surgical strategies. Patients undergo clinical auxiliary examinations, encompassing both imaging studies and laboratory analyses, to diagnose complications and medical histories. In this study, continuous variables included age, BMI, preoperative waiting time, surgical duration, intraoperative bleeding, length of stay, TP, PLT, WBC, and D-dimer levels. The remaining variables were categorized as binary inputs.

### 2.2 Statistical analysis

In Tables 1–3 of this study, we conducted inter-group comparisons for categorical variables using the chi-square test or Fisher’s exact test, with numerical values and percentages (%) employed to express categorical variables. Subsequently, for continuous variables, we initially assessed whether they adhered to a normal distribution. In cases where continuous variables exhibited a normal distribution, group comparisons were conducted using the mean with the addition and subtraction of the standard deviation (SD), and independent sample *t*-tests were employed. For continuous variables deviating from a normal distribution, descriptive statistics, including the median (interquartile range), were utilized, and inter-group differences were examined through non-parametric tests.

**Table 1:** Baseline clinical characteristics of the entire cohort of elderly hip fracture patients overall, in the PLOS and no-PLOS groups.

Variables	Total (*n* = 360)	No-PLOS (*n* = 257)	PLOS (*n* = 103)	*p*
Demographics				
Gender, *n* (%)				0.477
Female	239 (66)	174 (68)	65 (63)	
Male	121 (34)	83 (32)	38 (37)	
Age, years, Median (Q1,Q3)	81 (71, 87)	81 (70, 87)	82 (73.5, 87)	0.184
BMI, Median (Q1,Q3)	21.81 (19.56, 24.03)	21.77 (19.59, 24.17)	22.01 (19.33, 23.93)	0.782
Fracture-related				
Time from injury to admission, days, Median (Q1,Q3)	1 (0.5, 4)	1 (0.5, 3)	3 (1, 8)	<0.001
High energy traumame chanism, *n* (%)				0.208
No	347 (96)	250 (97)	97 (94)	
Yes	13 (4)	7 (3)	6 (6)	
Multiple traumas, *n* (%)				0.498
No	329 (91)	237 (92)	92 (89)	
Yes	31 (9)	20 (8)	11 (11)	
Preoperative immobilization, *n* (%)				0.773
No	240 (67)	173 (67)	67 (65)	
Yes	120 (33)	84 (33)	36 (35)	
Typeoffracture, *n* (%)				0.548
Extra-articular fracture	189 (52)	138 (54)	51 (50)	
Intra-articular fracture	171 (48)	119 (46)	52 (50)	
Surgery-related				
Typeofsurgery, *n* (%)				<0.001
Internal fixation	269 (75)	211 (82)	58 (56)	
Hip arthroplasty	91 (25)	46 (18)	45 (44)	
Preoperative waiting time, days, Median (Q1, Q3)	5 (3, 7)	4 (3, 5)	7 (6, 10)	<0.001
Duration of surgery, days, Median (Q1, Q3)	53 (46, 67)	53 (45, 65)	56 (49, 67.5)	0.022
Intraoperative bleeding, *n* (%)				0.733
50	12 (3)	8 (3)	4 (4)	
150	103 (29)	76 (30)	27 (26)	
200	177 (49)	121 (47)	56 (54)	
250	55 (15)	42 (16)	13 (13)	
300	13 (4)	10 (4)	3 (3)	
Blood transfusion, *n* (%)				0.002
No	343 (95)	251 (98)	92 (89)	
Yes	17 (5)	6 (2)	11 (11)	
Medical history				
Smoking, *n* (%)				0.503
No	235 (65)	171 (67)	64 (62)	
Yes	125 (35)	86 (33)	39 (38)	
Drinking, *n* (%)				0.653
No	290 (81)	205 (80)	85 (83)	
Yes	70 (19)	52 (20)	18 (17)	
Osteoporosis, *n* (%)				1
No	93 (26)	66 (26)	27 (26)	
Yes	267 (74)	191 (74)	76 (74)	
Lower extremity vascular disease, *n* (%)				<0.001
No	260 (72)	213 (83)	47 (46)	
Yes	100 (28)	44 (17)	56 (54)	
Diabetes, *n* (%)				0.196
No	280 (78)	205 (80)	75 (73)	
Yes	80 (22)	52 (20)	28 (27)	
Hypertension, *n* (%)				0.689
No	202 (56)	142 (55)	60 (58)	
Yes	158 (44)	115 (45)	43 (42)	
Alzheimer’s disease or dementia, *n* (%)				0.237
No	346 (96)	249 (97)	97 (94)	
Yes	14 (4)	8 (3)	6 (6)	
Heart failure, *n* (%)				0.002
No	345 (96)	252 (98)	93 (90)	
Yes	15 (4)	5 (2)	10 (10)	
Cardiovascular disease, *n* (%)				0.021
No	285 (79)	212 (82)	73 (71)	
Yes	75 (21)	45 (18)	30 (29)	
COPD or pulmonary fibrosis, *n* (%)				0.726
No	320 (89)	227 (88)	93 (90)	
Yes	40 (11)	30 (12)	10 (10)	
Cerebrovascular disease, *n* (%)				<0.001
No	322 (89)	240 (93)	82 (80)	
Yes	38 (11)	17 (7)	21 (20)	
Parkinson’s disease, *n* (%)				0.283
No	351 (98)	252 (98)	99 (96)	
Yes	9 (2)	5 (2)	4 (4)	
Malignanttumors, *n* (%)				0.359
No	354 (98)	254 (99)	100 (97)	
Yes	6 (2)	3 (1)	3 (3)	
Hemiplegia, *n* (%)				0.007
No	350 (97)	254 (99)	96 (93)	
Yes	10 (3)	3 (1)	7 (7)	
Laboratory examination				
Anemia, *n* (%)				<0.001
No	250 (69)	200 (78)	50 (49)	
Yes	110 (31)	57 (22)	53 (51)	
Hypoproteinemia, *n* (%)				<0.001
No	264 (73)	213 (83)	51 (50)	
Yes	96 (27)	44 (17)	52 (50)	
Renalinsufficiency, *n* (%)				<0.001
No	328 (91)	243 (95)	85 (83)	
Yes	32 (9)	14 (5)	18 (17)	
Electrolytedisorders, *n* (%)				<0.001
No	309 (86)	238 (93)	71 (69)	
Yes	51 (14)	19 (7)	32 (31)	
Liverdysfunction, *n* (%)				0.015
No	345 (96)	251 (98)	94 (91)	
Yes	15 (4)	6 (2)	9 (9)	
Arrhythmia, *n* (%)				0.132
No	320 (89)	233 (91)	87 (84)	
Yes	40 (11)	24 (9)	16 (16)	
Hyperlipidemia, *n* (%)				0.414
No	314 (87)	227 (88)	87 (84)	
Yes	46 (13)	30 (12)	16 (16)	
TP (g/dL), Median (Q1, Q3)	61.85 (58.38, 66.6)	61.6 (58.2, 66.6)	62.6 (59.05, 67.15)	0.155
PLT (×10^9/L), Median (Q1, Q3)	168 (127.75, 221)	168 (125, 219)	168 (132, 225)	0.754
WBC (×10^9/L), Median (Q1, Q3)	7.67 (6.02, 9.54)	7.37 (5.97, 9.02)	8.21 (6.26, 13.34)	<0.001
D-dimer (mg/L FEU), Median (Q1, Q3)	6.32 (3.34, 13.99)	4.99 (3.09, 11.13)	8.36 (5.02, 14.51)	<0.001

Median (Q1, Q3): the median (quartiles) was used for continuous variables that did not fit the normal distribution. Mean ± standard deviation: mean (positive and negative standard deviation, SD) was used for continuous variables that conformed to a normal distribution. *n* (%): categorical variables were expressed as numbers and percentages (%). COPD, chronic obstructive pulmonary disease; TP, total protein; PLT, platelets; WBC, white blood cells.

**Table 2:** Baseline clinical characteristics of the overall, PLOS and no-PLOS groups of the training set of elderly hip fracture patients.

Variables	Train (*n* = 253)	No-PLOS (*n* = 180)	PLOS (*n* = 73)	*p*
Demographics				
Gender, *n* (%)				0.121
Female	169 (67)	126 (70)	43 (59)	
Male	84 (33)	54 (30)	30 (41)	
Age, years, Median (Q1, Q3)	81 (71, 87)	81 (70, 87.25)	82 (74, 87)	0.298
BMI, Median (Q1, Q3)	21.56 (19.49, 24)	21.55 (19.56, 24.46)	21.61 (19.27, 23.44)	0.431
Fracture-related				
Time from injury to admission, days, Median (Q1, Q3)	1 (0.5, 4)	1 (0.5, 3)	3 (1, 8)	<0.001
High energy traumame chanism, *n* (%)				0.285
No	244 (96)	175 (97)	69 (95)	
Yes	9 (4)	5 (3)	4 (5)	
Multiple traumas, *n* (%)				0.22
No	232 (92)	168 (93)	64 (88)	
Yes	21 (8)	12 (7)	9 (12)	
Preoperative immobilization, *n* (%)				1
No	174 (69)	124 (69)	50 (68)	
Yes	79 (31)	56 (31)	23 (32)	
Typeoffracture, *n* (%)				0.686
Extra-articular fracture	135 (53)	98 (54)	37 (51)	
Intra-articular fracture	118 (47)	82 (46)	36 (49)	
Surgery-related				
Typeofsurgery, *n* (%)				<0.001
Internal fixation	188 (74)	149 (83)	39 (53)	
Hip arthroplasty	65 (26)	31 (17)	34 (47)	
Duration of surgery, days, Median (Q1, Q3)	53 (46, 67)	53 (46, 65)	56 (47, 68)	0.054
Intraoperative bleeding, *n* (%)				0.896
50	10 (4)	7 (4)	3 (4)	
150	72 (28)	51 (28)	21 (29)	
200	126 (50)	87 (48)	39 (53)	
250	36 (14)	28 (16)	8 (11)	
300	9 (4)	7 (4)	2 (3)	
Blood transfusion, *n* (%)				0.057
No	240 (95)	174 (97)	66 (90)	
Yes	13 (5)	6 (3)	7 (10)	
Medical history				
Smoking, *n* (%)				0.144
No	168 (66)	125 (69)	43 (59)	
Yes	85 (34)	55 (31)	30 (41)	
Drinking, *n* (%)				0.502
No	203 (80)	142 (79)	61 (84)	
Yes	50 (20)	38 (21)	12 (16)	
Osteoporosis, *n* (%)				0.783
No	68 (27)	47 (26)	21 (29)	
Yes	185 (73)	133 (74)	52 (71)	
Lower extremity vascular disease, *n* (%)				<0.001
No	180 (71)	149 (83)	31 (42)	
Yes	73 (29)	31 (17)	42 (58)	
Diabetes, *n* (%)				0.147
No	197 (78)	145 (81)	52 (71)	
Yes	56 (22)	35 (19)	21 (29)	
Hypertension, *n* (%)				0.669
No	142 (56)	99 (55)	43 (59)	
Yes	111 (44)	81 (45)	30 (41)	
Alzheimer’s disease or dementia, *n* (%)				0.158
No	243 (96)	175 (97)	68 (93)	
Yes	10 (4)	5 (3)	5 (7)	
Heart failure, *n* (%)				0.008
No	245 (97)	178 (99)	67 (92)	
Yes	8 (3)	2 (1)	6 (8)	
Cardiovascular disease, *n* (%)				0.026
No	195 (77)	146 (81)	49 (67)	
Yes	58 (23)	34 (19)	24 (33)	
COPD or pulmonary fibrosis, *n* (%)				0.999
No	227 (90)	161 (89)	66 (90)	
Yes	26 (10)	19 (11)	7 (10)	
Cerebrovascular disease, *n* (%)				<0.001
No	227 (90)	170 (94)	57 (78)	
Yes	26 (10)	10 (6)	16 (22)	
Parkinson’s disease, *n* (%)				0.628
No	248 (98)	177 (98)	71 (97)	
Yes	5 (2)	3 (2)	2 (3)	
Malignanttumors, *n* (%)				0.146
No	248 (98)	178 (99)	70 (96)	
Yes	5 (2)	2 (1)	3 (4)	
Hemiplegia, *n* (%)				0.023
No	246 (97)	178 (99)	68 (93)	
Yes	7 (3)	2 (1)	5 (7)	
Laboratory examination				
Anemia, *n* (%)				<0.001
No	183 (72)	145 (81)	38 (52)	
Yes	70 (28)	35 (19)	35 (48)	
Hypoproteinemia, *n* (%)				<0.001
No	186 (74)	150 (83)	36 (49)	
Yes	67 (26)	30 (17)	37 (51)	
Renalinsufficiency, *n* (%)				0.019
No	230 (91)	169 (94)	61 (84)	
Yes	23 (9)	11 (6)	12 (16)	
Electrolytedisorders, *n* (%)				<0.001
No	216 (85)	167 (93)	49 (67)	
Yes	37 (15)	13 (7)	24 (33)	
Liverdysfunction, *n* (%)				0.11
No	241 (95)	174 (97)	67 (92)	
Yes	12 (5)	6 (3)	6 (8)	
Arrhythmia, *n* (%)				0.456
No	229 (91)	165 (92)	64 (88)	
Yes	24 (9)	15 (8)	9 (12)	
Hyperlipidemia, *n* (%)				0.76
No	221 (87)	156 (87)	65 (89)	
Yes	32 (13)	24 (13)	8 (11)	
TP (g/dL), Median (Q1, Q3)	61.9 (58.6, 66.8)	61.75 (57.95, 66.73)	62.9 (60.1, 67.2)	0.103
PLT (×10^9/L), Median (Q1, Q3)	170 (131, 223)	169 (123.75, 221.25)	172 (149, 228)	0.449
WBC (×10^9/L), Median (Q1, Q3)	7.67 (5.97, 9.55)	7.44 (6.02, 9.03)	8.21 (5.76, 13.4)	0.018
D-dimer (mg/L FEU), Median (Q1, Q3)	6.38 (3.36, 12.68)	6.23 (3.08, 10.87)	7.09 (5, 14.53)	0.001

Median (Q1, Q3): the median (quartiles) was used for continuous variables that did not fit the normal distribution. Mean ± standard deviation: mean (positive and negative standard deviation, SD) was used for continuous variables that conformed to a normal distribution. *n* (%): categorical variables were expressed as numbers and percentages (%). COPD, chronic obstructive pulmonary disease; TP, total protein; PLT, platelets; WBC, white blood cells.

**Table 3:** Baseline clinical characteristics of the overall, PLOS, and no-PLOS groups of the test set of elderly hip fracture patients.

Variables	Test (*n* = 107)	No-PLOS (*n* = 77)	PLOS (*n* = 30)	*p*
Demographics				
Gender, *n* (%)				0.396
Female	70 (65)	48 (62)	22 (73)	
Male	37 (35)	29 (38)	8 (27)	
Age, years, Median (Q1, Q3)	79 (70, 85.5)	78 (70, 84)	79 (71.5, 86.5)	0.421
BMI, Mean ± SD	22.15 ± 3.55	21.87 ± 3.3	22.87 ± 4.08	0.24
Fracture-related				
Time from injury to admission, days, Median (Q1, Q3)	56 (45.5, 67)	53 (45, 67)	56.5 (51.25, 67)	0.222
High energy traumame chanism, *n* (%)				0.313
No	103 (96)	75 (97)	28 (93)	
Yes	4 (4)	2 (3)	2 (7)	
Multiple traumas, *n* (%)				0.722
No	97 (91)	69 (90)	28 (93)	
Yes	10 (9)	8 (10)	2 (7)	
Preoperative immobilization, *n* (%)				0.656
No	66 (62)	49 (64)	17 (57)	
Yes	41 (38)	28 (36)	13 (43)	
Typeoffracture, *n* (%)				0.692
Extra-articular fracture	55 (51)	41 (53)	14 (47)	
Intra-articular fracture	52 (49)	36 (47)	16 (53)	
Surgery-related				
Typeofsurgery, *n* (%)				0.107
Internal fixation	81 (76)	62 (81)	19 (63)	
Hip arthroplasty	26 (24)	15 (19)	11 (37)	
Duration of surgery, days, Median (Q1, Q3)				
Intraoperative bleeding, *n* (%)				0.552
50	2 (2)	1 (1)	1 (3)	
150	31 (29)	25 (32)	6 (20)	
200	51 (48)	34 (44)	17 (57)	
250	19 (18)	14 (18)	5 (17)	
300				
Blood transfusion, *n* (%)				0.005
No	103 (96)	77 (100)	26 (87)	
Yes	4 (4)	0 (0)	4 (13)	
Medical history				
Smoking, *n* (%)				0.446
No	67 (63)	46 (60)	21 (70)	
Yes	40 (37)	31 (40)	9 (30)	
Drinking, *n* (%)				1
No	87 (81)	63 (82)	24 (80)	
Yes	20 (19)	14 (18)	6 (20)	
Osteoporosis, *n* (%)				0.796
No	25 (23)	19 (25)	6 (20)	
Yes	82 (77)	58 (75)	24 (80)	
Lower extremity vascular disease, *n* (%)				0.003
No	80 (75)	64 (83)	16 (53)	
Yes	27 (25)	13 (17)	14 (47)	
Diabetes, *n* (%)				1
No	83 (78)	60 (78)	23 (77)	
Yes	24 (22)	17 (22)	7 (23)	
Hypertension, *n* (%)				1
No	60 (56)	43 (56)	17 (57)	
Yes	47 (44)	34 (44)	13 (43)	
Alzheimer’s disease or dementia, *n* (%)				1
No	103 (96)	74 (96)	29 (97)	
Yes	4 (4)	3 (4)	1 (3)	
Heart failure, *n* (%)				0.095
No	100 (93)	74 (96)	26 (87)	
Yes	7 (7)	3 (4)	4 (13)	
Cardiovascular disease, *n* (%)				0.557
No	90 (84)	66 (86)	24 (80)	
Yes	17 (16)	11 (14)	6 (20)	
COPD or pulmonary fibrosis, *n* (%)				0.753
No	93 (87)	66 (86)	27 (90)	
Yes	14 (13)	11 (14)	3 (10)	
Cerebrovascular disease, *n* (%)				0.311
No	95 (89)	70 (91)	25 (83)	
Yes	12 (11)	7 (9)	5 (17)	
Parkinson’s disease, *n* (%)				0.313
No	103 (96)	75 (97)	28 (93)	
Yes	4 (4)	2 (3)	2 (7)	
Malignanttumors, *n* (%)				1
No	106 (99)	76 (99)	30 (100)	
Yes	1 (1)	1 (1)	0 (0)	
Hemiplegia, *n* (%)				0.189
No	104 (97)	76 (99)	28 (93)	
Yes	3 (3)	1 (1)	2 (7)	
Laboratory examination				
Anemia, *n* (%)				0.005
No	67 (63)	55 (71)	12 (40)	
Yes	40 (37)	22 (29)	18 (60)	
Hypoproteinemia, *n* (%)				0.002
No	78 (73)	63 (82)	15 (50)	
Yes	29 (27)	14 (18)	15 (50)	
Renalinsufficiency, *n* (%)				0.014
No	98 (92)	74 (96)	24 (80)	
Yes	9 (8)	3 (4)	6 (20)	
Electrolytedisorders, *n* (%)				0.021
No	93 (87)	71 (92)	22 (73)	
Yes	14 (13)	6 (8)	8 (27)	
Liverdysfunction, *n* (%)				0.02
No	104 (97)	77 (100)	27 (90)	
Yes	3 (3)	0 (0)	3 (10)	
Arrhythmia, *n* (%)				0.142
No	91 (85)	68 (88)	23 (77)	
Yes	16 (15)	9 (12)	7 (23)	
Hyperlipidemia, *n* (%)				0.021
No	93 (87)	71 (92)	22 (73)	
Yes	14 (13)	6 (8)	8 (27)	
TP (g/dL), Mean ± SD	61.97 ± 6.48	61.88 ± 6.36	62.18 ± 6.87	0.841
PLT (×10^9/L), Median (Q1, Q3)	155 (121.5, 207.5)	159 (127, 210)	149 (112, 189.75)	0.464
WBC (×10^9/L), Median (Q1, Q3)	7.67 (6.17, 9.48)	7.33 (5.79, 8.92)	8.3 (7.35, 11)	0.013
D-dimer (mg/L FEU), Median (Q1, Q3)	6.15 (3.22, 14.46)	4.77 (3.11, 11.83)	12.54 (5.11, 14.48)	0.013

Median (Q1, Q3): the median (quartiles) was used for continuous variables that did not fit the normal distribution. Mean ± standard deviation: mean (positive and negative standard deviation, SD) was used for continuous variables that conformed to a normal distribution. *n* (%): categorical variables were expressed as numbers and percentages (%). COPD, chronic obstructive pulmonary disease; TP, total protein; PLT, platelets; WBC, white blood cells.

The Random Forest algorithm utilized multiple decision trees for prediction, falling under the umbrella of ensemble learning methodologies. Through a bootstrapping process, each decision tree was trained on a subset of the data, introducing diversity among the trees. During the training process, each split was corresponded to a random subset of features, a characteristic that aids in preventing overfitting and ensuring the significance of all features in the model. This contributed to a more robust and accurate overall prediction of the model. The entire patient cohort was randomly divided into training and testing sets in a 70:30 ratio. We used the randomly acquired training set to construct the Random Forest predictive model. The Bootstrapping resampling/Bagging method involved the random extraction and replacement of data to create multiple training sample sets, each undergoing individual decision tree training. Due to the inherent nature of the Bootstrapping method, which may result in unsampled data, it is aptly referred to as out-of-bag (OOB). Utilizing this subset of data to derive the OOB error rate served as a means to assess the generalization capability of the constructed model. Each decision tree within the Random Forest ensemble contributed its “vote” to the PLOS outcomes, with the aggregation of these votes culminating in the ultimate predictive result. Feature selection was orchestrated through sophisticated techniques of “feature bagging” or “attribute bagging,” a pivotal step in the realm of the Random Forest algorithm. This process obtained crucial variables, ranking them based on the average reduction rates of accuracy and Gini coefficients. Subsequently, these vital variables were employed to craft the requisite predictive model. The Random Forest predictive model in question was trained with 500 decision trees, each node of which randomly selected four variables.

We subjected the data from both the training and testing datasets to scrutiny using the completed Random Forest algorithm model. Simultaneously, we constructed a Lasso regression model, examining the data from both the training and testing datasets. The resultant outcomes were further juxtaposed against the Random Forest predictive model. It is widely acknowledged that Lasso regression introduces L1 regularization, serving as a preventive measure against overfitting. When the *λ* (lambda) parameter was sufficiently large, it shrunk certain coefficients to zero, encouraging sparsity and facilitating the identification of the most influential features. Determining the optimal penalty coefficient *λ* for the Lasso regression model involved selecting the value that minimizes the error plus one standard error using a 10-fold cross-validation approach. This method ensures robustness in model selection and parameter tuning. The Lasso regression model, with its selected features and assigned non-zero coefficients, allows for the interpretation and examination of the relative importance of variables screened by the Random Forest. Subsequently, the model was validated from various perspectives. The AUC was computed for both the Random Forest algorithm model and the Lasso regression model, followed by a comparative analysis. The Random Forest predictive model underwent validation on the training and testing datasets, assessing accuracy through Balanced Accuracy, and examining the model’s reliability using Kappa statistics and F1 scores.

In this study, statistical analyses were conducted using R Studio (R-4.3.2, 64-bit). The R packages employed encompassed caret, Boruta, randomForest, corrplot, ggplot2, and glmnet. The construction of the random forest predictive model was achieved through the utilization of the Boruta, caret, and randomforest packages. The Lasso regression model, on the other hand, was implemented using the glmnet package. Statistical significance was confirmed at a threshold of <0.05.

## 3 Results

### 3.1 Patient characteristics

**Figure 1:**
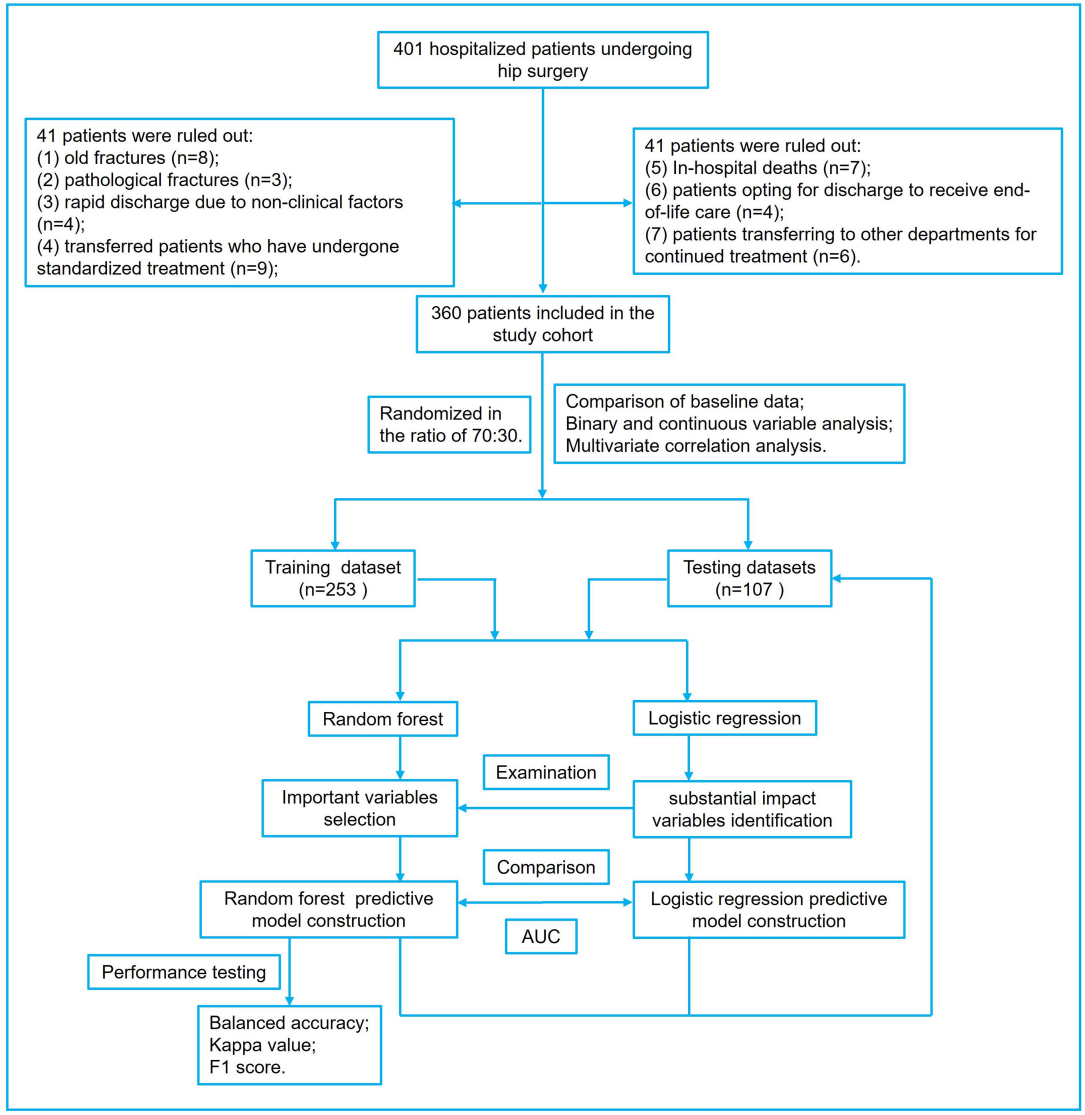
The figure presents the workflow for constructing the random forest prediction model for extended hospital stays in elderly hip fracture patients. Additionally, a Lasso regression prediction model was developed to validate and compare the random forest algorithm.

**Figure 2:**
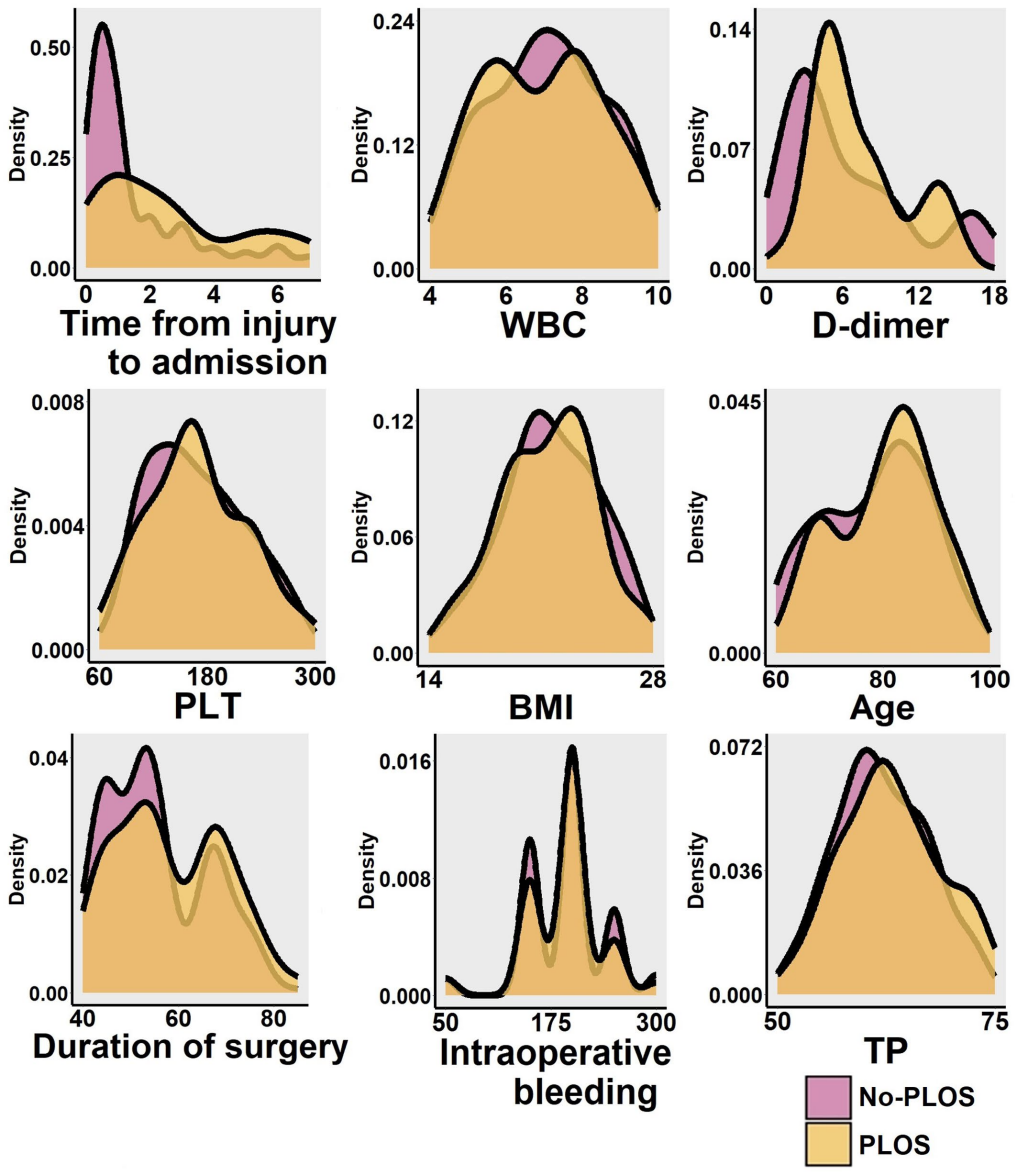
The study visually presents the distribution of continuous variables such as age, BMI, preoperative waiting time, surgical duration, intraoperative bleeding, length of stay, TP, PLT, WBC, and D-dimer levels. It also displays the results for both the PLOS group and the no-PLOS group for comparison.

**Figure 3:**
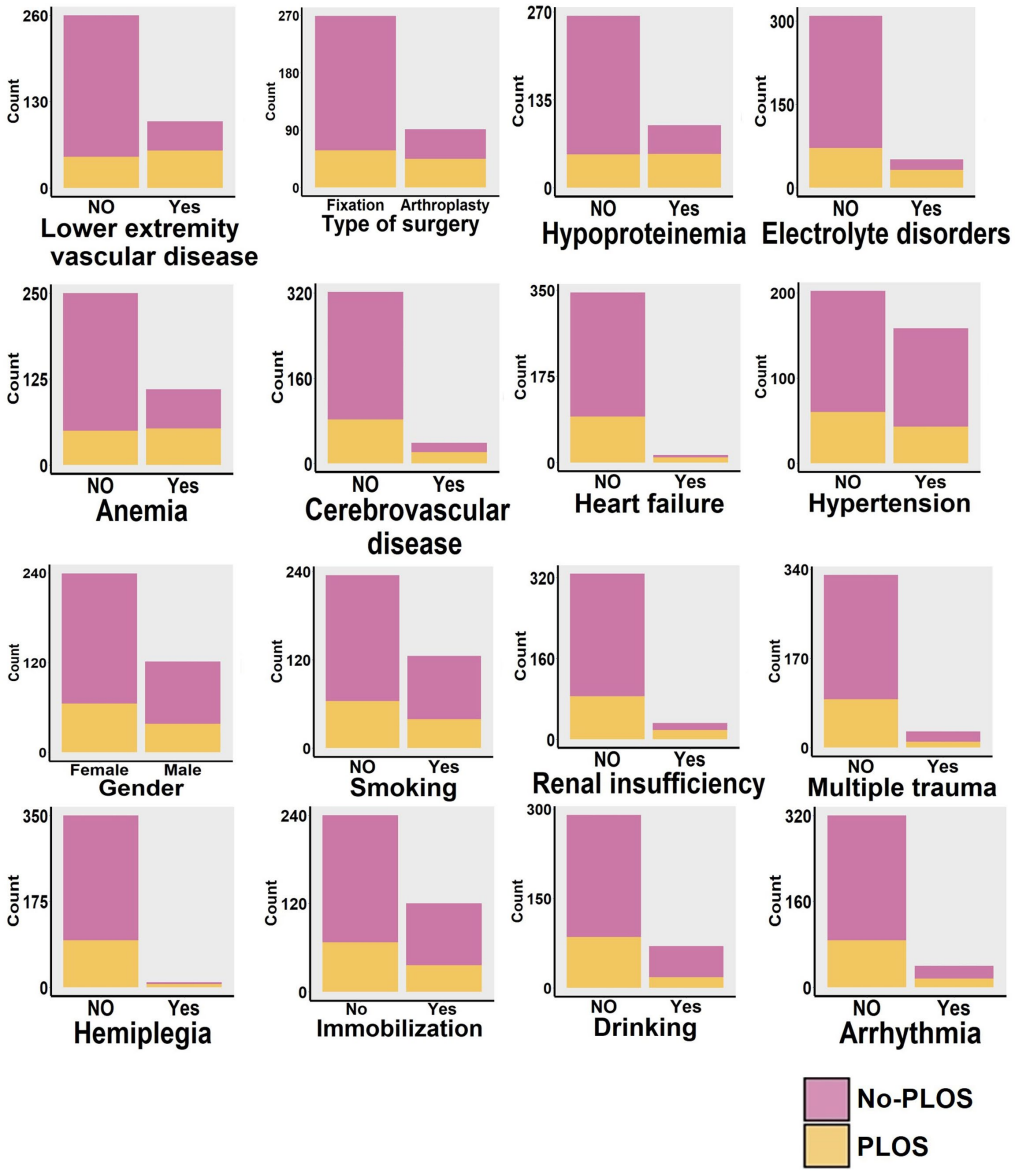
Based on the feature ranking from the random forest algorithm (illustrated in Figure 5), we identified the top 16 binary variables. These 16 binary variables were then subjected to visual analysis, comparing their distributions across the PLOS and no-PLOS groups.

**Figure 4:**
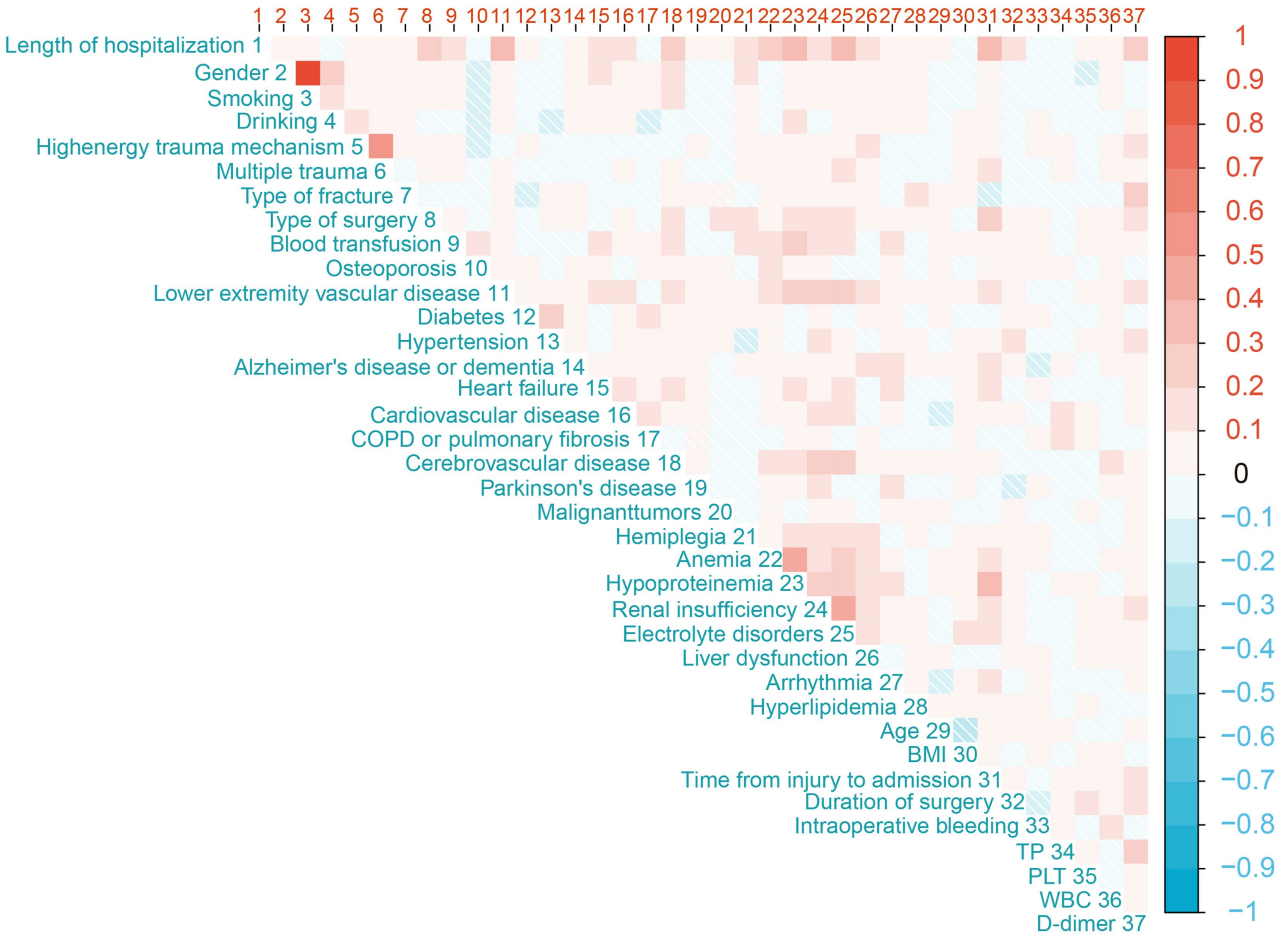
The figure presents a correlation analysis of all variables mentioned in this study, depicted as correlation heatmap.

This study presents the baseline characteristics of the entire patient cohort in Table 1. The establishment and validation of the Random Forest predictive model in this study are shown in Figure 1. The entire patient cohort underwent a random allocation into training and testing datasets, preserving a ratio of 70:30. The distinctive baseline characteristics of these cohorts are expounded upon in Tables 2, 3. The median age (interquartile range) of the entire patient cohort is 81 (71, 87) years, with 66.39% comprising 239 females. Among the selected 360 patients, the occurrence rate of PLOS, defined by the 75th percentile, is 28.61%. Visual representations of continuous variables in the PLOS and no-PLOS groups are illustrated in Figure 2. For binary variables, the top 16, ranked based on feature selection through the Random Forest algorithm, are visualized in Figure 3. Important variables identified through univariate analysis (*p* < 0.05) include Time from injury to admission (*p* < 0.001), Lower extremity vascular disease (*p* < 0.001), Preoperative waiting time (*p* < 0.001), Duration of surgery (*p* < 0.022), Blood transfusion (*p* < 0.002), Cardiovascular disease (*p* < 0.021), Cerebrovascular disease (*p* < 0.001), Hemiplegia (*p* < 0.007), Anemia (*p* < 0.001), Hypoproteinemia (*p* < 0.001), Renal insufficiency (*p* < 0.001), Electrolyte disorders (*p* < 0.001), Type of surgery (*p* < 0.001), Liver dysfunction (*p* < 0.015), WBC (*p* < 0.001), Heart failure (*p* < 0.002), and D-dimer (*p* < 0.001). Subsequently, a correlation analysis was conducted on all variables, and a correlation heatmap was prepared (Figure 4). During feature selection, post-admission preoperative waiting time was excluded because it was positively correlated with the total hospitalization period. Despite its exclusion, preoperative preparation time remains a crucial aspect for assessing the length of hospital stay, as it indicates the patient’s overall health and influences medical planning decisions.

### 3.2 Important variables selection

**Figure 5:**
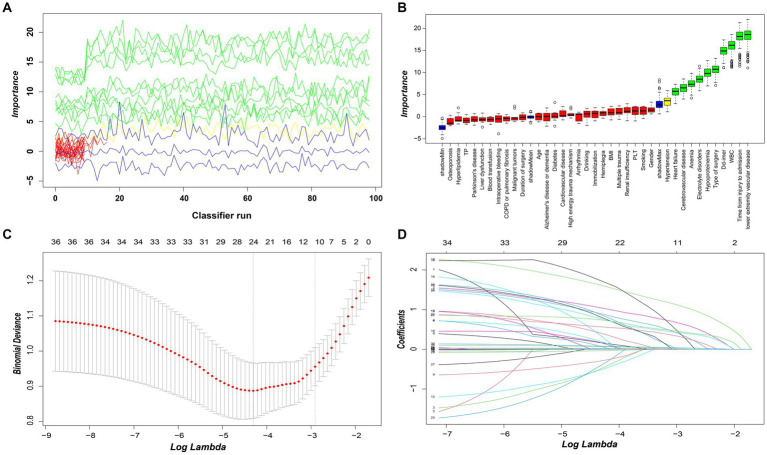
**(A)** The results of the decision to reject or accept features by the Random Forest algorithm using the Boruta function run 100 times. **(B)** Random forest algorithm for feature screening and importance ranking. The box-and-line plot shows the random forest algorithm features selected as important, tentative and unimportant variables in red, yellow and red, respectively. **(C)** The figure illustrates the Lasso regression model employing L1 regularization and robust shrinkage capabilities to perform feature selection. By using the plus one standard error and ten-fold cross-validation criterion, the optimal tuning parameter *λ* was determined to identify the variables with the most significant impact (10 variables of major influence). **(D)** The figure shows the distribution of Lasso coefficients for the feature variables in this study.

**Figure 6:**
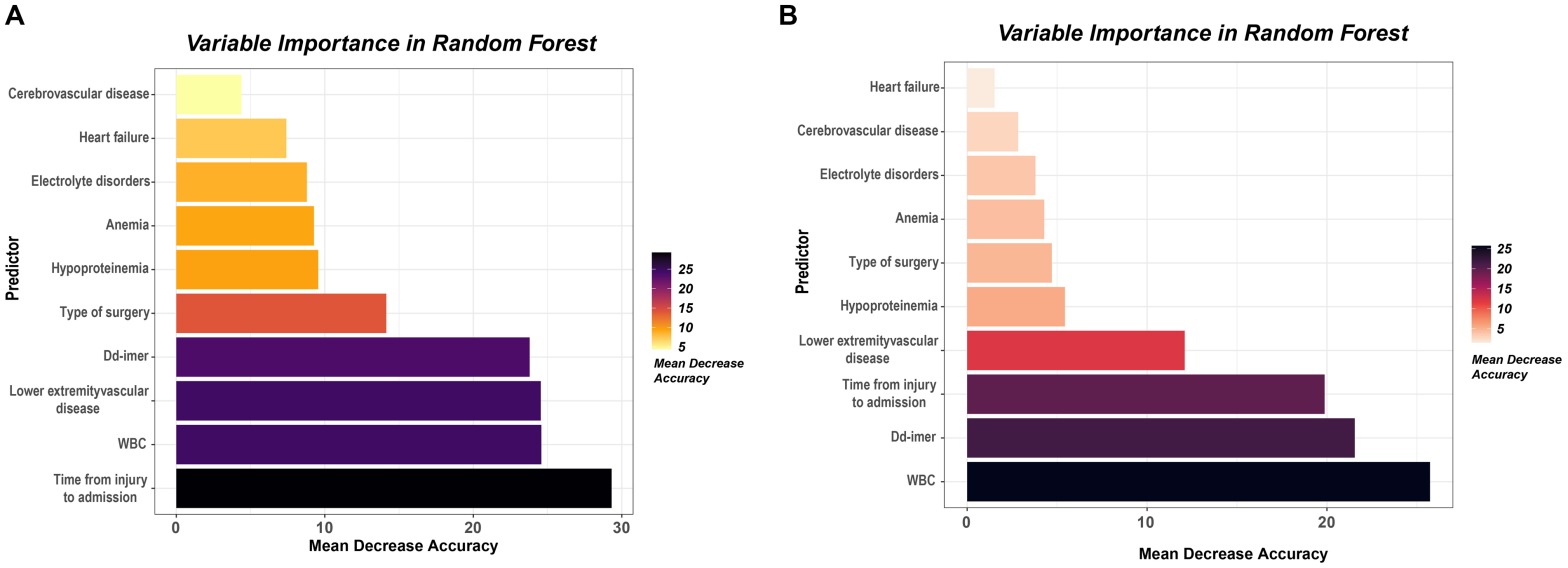
**(A)** The 10 variables selected using the metrics mean decrease accuracy. These variables have been ordered according to their importance. **(B)** The 10 variables selected using the metrics mean decrease Gini. These variables have been ordered according to their importance.

Upon inputting all variables, the commencement of feature selection emerged as the pivotal stride in constructing the random forest predictive model. The algorithm orchestrates the meticulous curation and prioritization of features, culminating in the process and outcomes delineated in Figures 5A,B. The findings unequivocally identified ten pivotal variables, encompassing lower extremity vascular disease, time from injury to admission, WBC, D-dimer, type of surgery, hypoproteinemia, electrolyte disorders, anemia, cerebrovascular disease, and heart failure. Subsequently, this inquiry undertaken feature selection through Lasso regression, aiming to substantiate and ascertain the relative significance of the results gleaned from random forest feature selection. Leveraging L1 regularization and robust shrinkage capabilities, Lasso regression discerned variables of substantial influence. Figures 5C,D showcase the outcomes of Lasso regression, unveiling the most influential variables, predominantly encompassing the type of surgery, lower extremity vascular disease, heart failure, cerebrovascular disease, anemia, hypoproteinemia, electrolyte disorders, time from injury to admission, WBC, and D-dimer. Notably, these variables exhibit a complete overlap with the previously established crucial variables in random forest analysis. This attests to the discernment of robust and reliable features, augmenting the interpretability and generalizability of the predictive model, while mitigating the risk of overfitting. Figure 6 illustrates the hierarchical arrangement of important variables generated by the Random Forest algorithm, showcasing their sorting based on Mean Decrease Accuracy and Mean Decrease Gini.

### 3.3 Random forest prediction model performance tests

**Figure 7:**
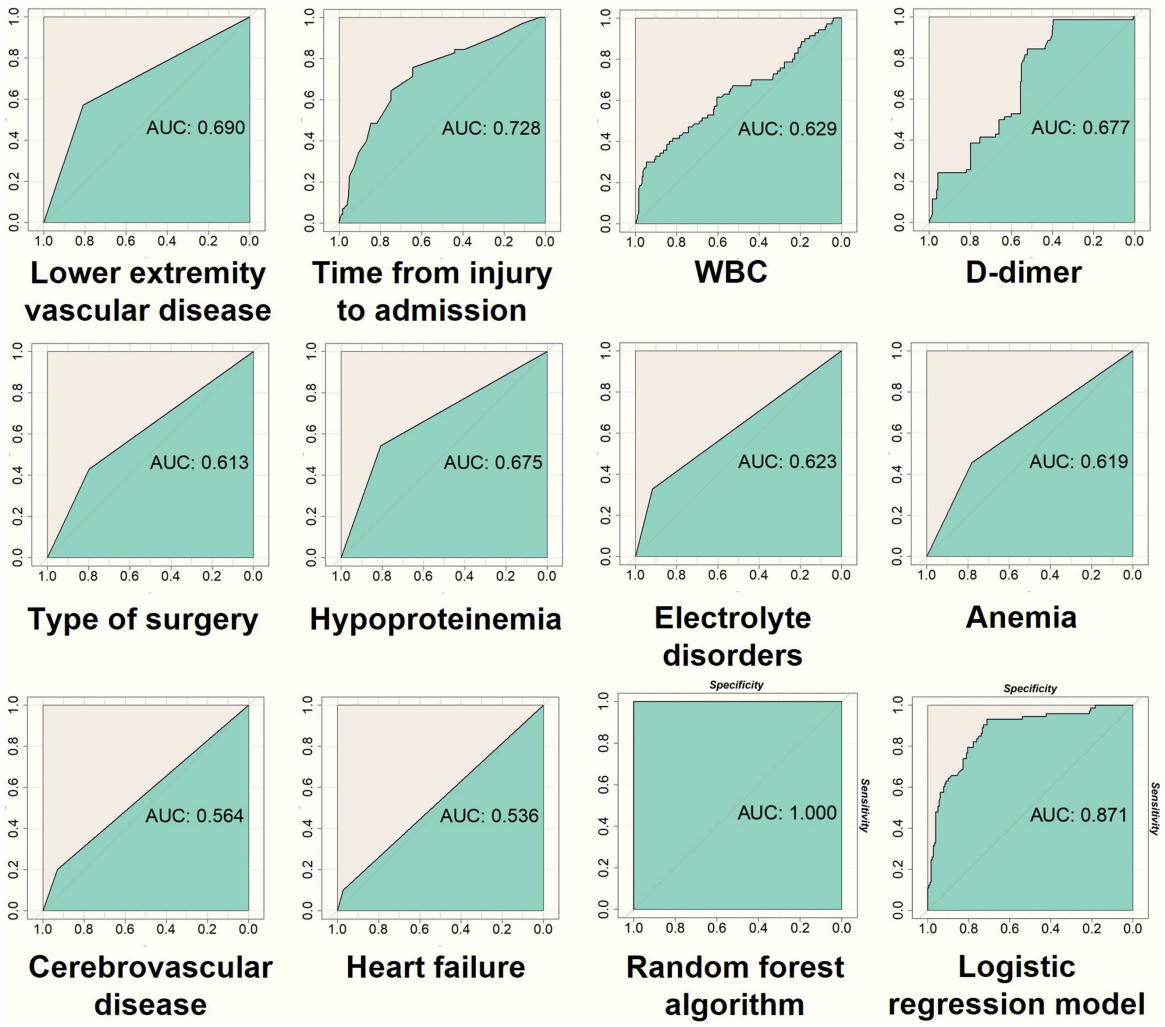
In the training dataset, the 10 most important variables selected through feature selection are used to generate ROC curves (as depicted in the first 10 figures). The final two figures in the bottom right corner illustrate the ROC curves obtained from the prediction models of the random forest and Lasso regression on the training data. These visualizations assist in evaluating the discriminatory performance of both models.

**Figure 8:**
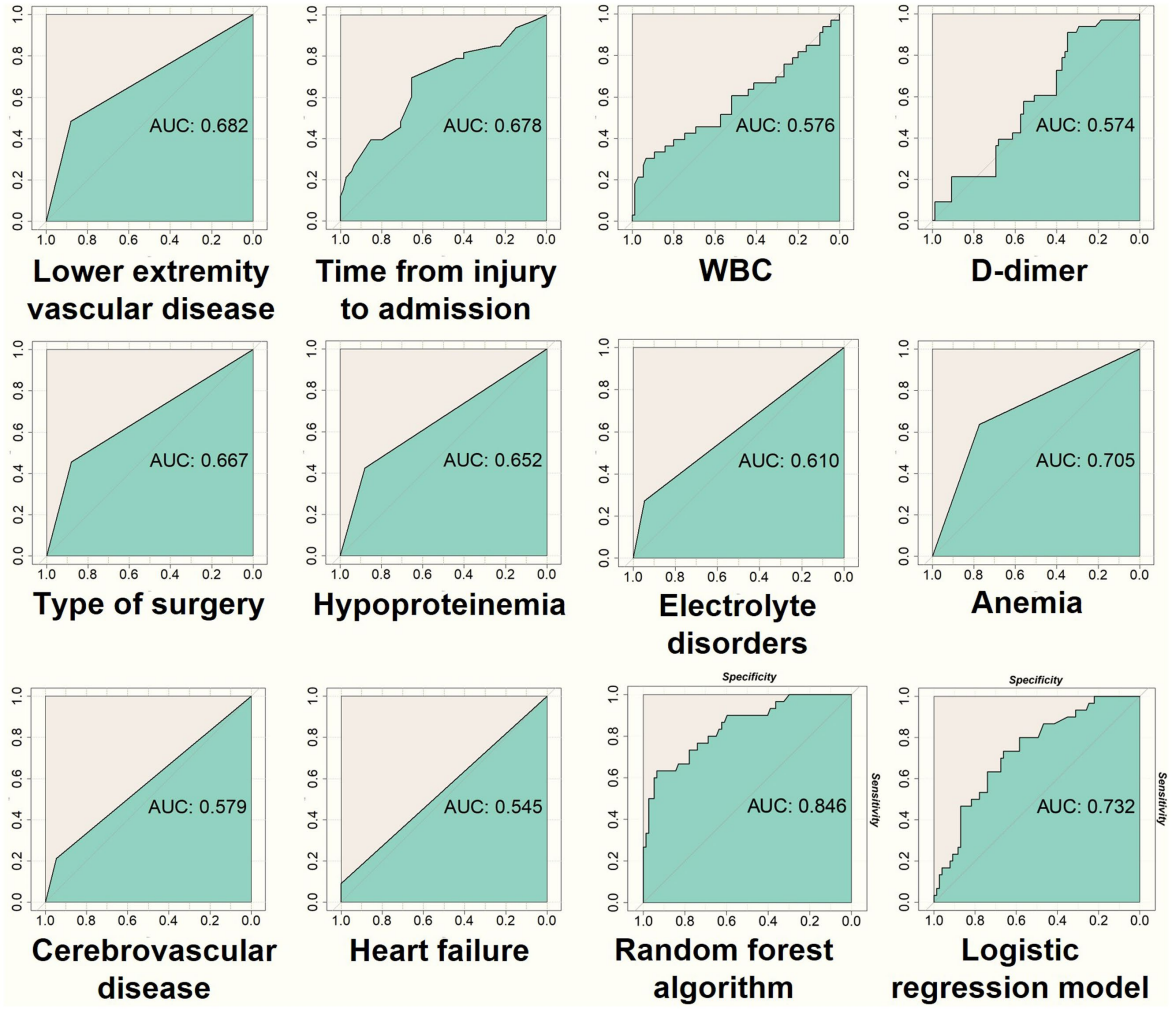
The figure presents ROC curves generated using the testing dataset, following a similar process as in Figure 7. It evaluates the discriminatory performance of the random forest and Lasso regression prediction models.

Founded upon the discernment of ten verified pivotal variables utilizing the random forest algorithm, this study culminates in the construction of a predictive model. This meticulously crafted model comprises 500 decision trees, each attempt at variable splitting involving four variables. Subsequently, the Out-of-Bag (OOB) assessment of the error rate served as a metric for assessing the model’s generalization capacity, revealing a remarkably diminutive OOB of 12.25%, signifying minimal generalization error. Model validation occurs through the utilization of both the training dataset, instrumental in constructing the model, and a distinct testing dataset (Figures 7, 8). The AUC for the training dataset attained perfection at 1.000, while the testing dataset registered a commendable 0.846. In the realm of scrutinizing discriminative prowess, this study also introduced a Lasso logistic regression predictive model. The AUC resulted for the training and testing groups stand at 0.871 and 0.732, attesting to the commendable discriminative acumen of the random forest predictive model. In this study, the random forest predictive model showcased flawless performance on the training dataset, achieving balanced accuracy, Kappa value, and F1 score, each peaking at 1.000. Correspondingly, on the testing dataset, these metrics materialize as 0.7294, 0.4325, and 0.6061.

## 4 Discussion

Amid the growing aging population, the rise in hip fractures among elderly patients has become a critical concern in orthopedic science (4). Despite improvements in surgical approaches and faster postoperative recovery, extended hospitalization periods have substantial impacts on both patients and healthcare institutions. Prolonged hospital stays increase economic burdens, raise the risk of complications, and elevate mortality risks for patients (1). The mutual progression of complications and extended hospitalization highlights the interdependence of these factors (12). Moreover, prolonged hospital stays lead to inefficient use of healthcare resources, heighten the challenges faced by medical professionals in management, decrease hospital bed turnover rates, and lower patient satisfaction (13). To address this issue, we have developed a random forest predictive model to forecast and assess the factors associated with extended hospital stays for elderly hip fracture patients undergoing surgical intervention.

The “important variables” identified in this study have demonstrated their significance in other related works, further underscoring their value in clinical applications. In this study, lower extremity vascular disease includes thrombosis and arteriosclerosis in the lower limb, causing vascular occlusion or narrowing. Studies suggest a link between lower extremity vascular disease in elderly hip fracture surgical patients and extended hospital stays. Thrombosis and arteriosclerosis in the lower limbs lead to reduced mobility, prolonged hospitalization, and higher risks of infection, pressure ulcers, and pneumonia in elderly hip fracture patients (14). Similarly, patients with prolonged hospital stays after medical care are at risk of complications such as urinary tract infections, pneumonia, and deep vein thrombosis (15, 16). The study by Chen et al. (17), identifies hip fractures in elderly patients as potential factors for prolonged bed rest, which can lead to acute conditions such as stroke, pressure sores, lung infections, and deep vein thrombosis (DVT) in the lower limbs. These complications are noted as significant risk factors for mortality in elderly hip fracture patients. Feng et al.’s study on preoperative independent risk factors for DVT suggests that hospitalization may need to be appropriately extended for high-risk patients, with recommendations for proactive postoperative care, chemical prophylaxis (such as low molecular weight heparin), and physical therapy to reduce the risk of complications (18). Peripheral arterial occlusive disease (PAOD) is a leading manifestation of chronic arterial occlusive pathology, mainly resulting from atherosclerosis. Its clinical presentation is characterized by a gradual and progressive restriction of blood flow due to vascular constriction, obstruction, or spasm, leading to insufficient oxygen supply to tissues (19). Besides PAOD, anemia and hypoxia can increase the risk of ulceration. As symptoms worsen, patients may experience ulceration and necrosis in the lower extremities, leading to severe and persistent pain that can cause secondary infections, cardiovascular events, and toxemia. Research shows that antithrombotic therapy (such as low molecular weight heparin) can slow disease progression and decrease the incidence of cardiovascular events in elderly heart failure patients at risk of developing PAOD (20, 21). Research by Birişik et al. (22) indicates that preoperative detection of femoral artery calcification in hip fracture patients can be used as a radiological parameter and an independent predictor of mortality in elderly hip fracture patients. Similarly, Alison et al. (23) conducted a study that aligns treatment time and length of hospital stay (LOS) with the postoperative needs of hip fracture patients to optimize discharge outcomes and manage costs. The study demonstrates that LOS is linked to the functional capacity of patients undergoing postoperative rehabilitation and their ability to perform self-care tasks.

Numerous studies have shown that prompt surgical intervention after a fracture significantly reduces patient mortality. The time from fracture occurrence to hospital admission is a crucial factor in ensuring early surgery for elderly individuals with hip fractures. For example, Klestil et al. (24) found that performing surgery within 48 h for elderly hip fracture patients led to a 20% reduction in 1 year mortality and a lower rate of perioperative complications. Tulic et al. (25) noted that a preoperative stay exceeding 72 h increased the overall complication rate. This suggests that a shorter interval between hip fracture occurrence and hospital admission enables early admission and subsequent timely surgical intervention, which is essential for reducing complications and mortality risk. This, in turn, can influence the length of hospitalization. In choosing between internal fixation and joint arthroplasty, existing research emphasizes the importance of considering the patient’s overall health, type of fracture, and potential for postoperative functional recovery. While there is no clear direct correlation established in the literature, the selection between these surgical options can influence surgical outcomes, postoperative care, and rehabilitation approaches, which can help optimize the patient’s hospital stay duration. Ahmed’s et al. study, published in ScienceDirect, highlights that joint replacement surgery offers an advantageous approach for patients aged 80 and older with compromised bone quality who must adhere to weight-bearing restrictions (26). A comparative study assessing internal fixation, hemiarthroplasty, and total hip arthroplasty (THA) found that THA initially exhibited a higher incidence of complications (27). However, it showed positive results in both short-term and long-term postoperative periods, supporting early patient recovery and reducing hospital stay length.

In elderly patients undergoing hip fracture surgery, there is a link between cerebrovascular diseases, specifically acute cerebral infarction, and prolonged hospitalization. A study by Yuan et al. (28) examined patients with hip fractures who also experienced acute cerebral infarction. The findings reveal a negative impact on patients’ neurological recovery, leading to extended hospital stays, increased complication rates, and higher 1 year mortality rates. Despite these challenges, surgical intervention can enhance the prognosis for patients with hip fractures and acute cerebral infarction. Lim et al. (29) discuss the heightened risk associated with surgical intervention for hip fractures in patients who also have a stroke, resulting in poor expected functional recovery, recurrent strokes, myocardial infarction, and even death. This indicates that a careful approach is needed when managing patients with both stroke and hip fractures, as the coexistence can lead to longer hospital stays and worsening complications. Espinosa et al. (30) explore the increased incidence of stroke in elderly patients following hip fractures, which can lead to extended hospitalization and increased treatment demands. Heart failure is a common clinical condition characterized by symptoms such as fatigue, dyspnea, reduced exercise capacity, hypotension, refractory volume overload, and poor organ perfusion. Cullen et al. (31) conducted groundbreaking research that examined the relationship between heart failure and postoperative hospitalization length after hip fractures. Their study found a significant increase in the average hospital stay for patients with a preoperative heart failure diagnosis. Research by Cha et al. (32) indicates that impaired left ventricular systolic function is the primary risk factor for higher early postoperative mortality rates in elderly hip fracture patients. In this patient group, the 30 day mortality rate is 4.01 times higher in cases of severe heart failure compared to mild heart failure. The study by You et al. (33) emphasizes several risk factors that contribute to the onset of postoperative heart failure, including hypertension, hypoalbuminemia, anemia, age ≥ 70 years, and surgery duration ≥120 min. This research highlights the importance of thoroughly understanding the factors that influence heart failure after surgery to improve patient outcomes and minimize risks.

Postoperative anemia following hip joint surgery can result in a higher incidence of complications and delayed functional recovery in patients, leading to prolonged hospital stays and increased healthcare costs (34). Current research largely investigates the impact of hemoglobin levels on interventions for elderly patients undergoing hip fracture surgery. The study by Cao et al. (21) suggests that anemia in elderly hip fracture patients significantly affects the length of hospital stay. This is mainly due to the negative impact of blood volume supplementation in anemic patients on immune and coagulation system functions. Moreover, severe postoperative anemia can impede patient recovery after surgery. Furthermore, Willems et al. (35) found that an increase in hemoglobin levels postoperatively in elderly hip fracture patients was associated with shorter hospital stays. However, they noted that preoperative hemoglobin levels did not show a clear correlation, warranting further exploration through future randomized clinical trials. In contrast, earlier research by Halm et al. (36) indicated that higher preoperative hemoglobin levels were linked to shorter hospital stays, as well as lower mortality rates and fewer rehospitalizations within 60 days after discharge. Their study also underscored a relationship between postoperative hemoglobin levels and hospitalization duration or rehospitalization rates. Treatment for anemic patients often involves medication such as erythropoiesis-stimulating agents and iron supplements, as well as blood transfusions.

There are four crucial variables representing the outcomes of laboratory examinations, including WBC, D-dimer, hypoproteinemia, and electrolyte disorders. WBC and D-dimer values are derived by averaging admission and on the first postoperative day blood test results to provide a comprehensive overview of patients’ conditions during hospitalization. Elevated WBC levels are typically indicative of infection and may also occur in response to autoimmune diseases, bone marrow dysfunction, stress, or trauma. In elderly patients who experience hip fractures or undergo surgery, an increase in WBC is a common response to trauma or surgery. According to Gregory et al. (37), in the absence of abnormal clinical signs and symptoms, a post-hip replacement surgery rise in WBC levels may not immediately necessitate further investigation for infection. However, Tang et al.’s findings suggest that preoperative elevation of white blood cells does not have a clear correlation with adverse postoperative outcomes for patients (38). This underscores the importance of monitoring postoperative changes in WBC levels and providing appropriate medical care. A rise in WBC levels postoperatively can also signal the possible onset of infection, requiring prompt attention and management. Research by Yu et al. (39) demonstrates that elderly patients with hip fractures face various postoperative risk factors such as diabetes, hypoalbuminemia, general anesthesia, and surgical duration ≥120 min, increasing the likelihood of pneumonia. Healthcare professionals can proactively address these risk factors. However, other factors like prolonged hospitalization, worsening nutritional status, and increased bedridden time may lead to opportunistic pulmonary infections (40). Current research on hip fractures in the elderly primarily identifies D-dimer as a crucial predictor of deep vein thrombosis (DVT), which may explain its relationship with extended hospital stays. The study by Tian et al. (11) suggests that elevated D-dimer levels are a key factor for predicting prolonged hospital stays in elderly hip fracture patients. This predictive approach, using machine learning, aids in the efficient allocation and utilization of medical resources. Hypoproteinemia and electrolyte disorders stem from diagnostic criteria based on laboratory findings. Post-diagnosis, both conditions exert a profound influence on the overall duration of hospitalization. Therefore, our inclusion criteria revolve around diagnoses made during hospitalization or persisting until discharge. In elderly hip surgery patients, the occurrence of hypoalbuminemia (≤35 g/L) signifies a slower postoperative recovery, diminished functional outcomes, and a lower quality of life (41). Additionally, postoperative hypoalbuminemia in elderly hip fracture patients increases the risk of postoperative respiratory failure (PRF), which is associated with respiratory infections and extended hospital stays (42). Research by Cho et al. (43) highlights the prevalence of inadequate protein intake among elderly hip fracture patients, which can result in decreased muscle strength. After hip fracture surgery, insufficient physical rehabilitation can slow postoperative recovery and increase the likelihood of complications. To address hypoalbuminemia, medical care should involve individualized plans for oral, enteral, or parenteral protein intake, aiming to quickly restore normal protein levels and support the recovery process. Advanced age in elderly hip fracture patients is a risk factor for fluid and electrolyte imbalances, which can lead to an increased risk of pre- and postoperative delirium, particularly when associated with low sodium and calcium levels (44). Prolonged postoperative electrolyte imbalance can negatively impact patient prognosis. A study by Rudge et al. (45) found that postoperative hyponatremia is common in traumatic hip fracture patients and is associated with extended hospital stays, suggesting that the surgical process itself may be a contributing risk factor. Kemal et al. (46) reported a 20.5% incidence of hyponatremia in hip fracture patients, with similar mortality rates compared to patients with normal sodium levels. However, these patients experienced longer hospital stays, emphasizing the need to carefully manage hyponatremia. Proper perioperative preparation for elderly hip surgery patients includes vigilant monitoring of electrolyte levels, early identification of imbalances, and prompt management to improve patient outcomes and reduce hospitalization duration.

The aim of this study is to develop a predictive computational model for assessing the likelihood of PLOS in elderly patients undergoing hip surgery. The study identified 10 critical variables during the construction of the random forest predictive model, including lower extremity vascular disease, time from injury to hospital admission, white blood cell count, D-dimer levels, type of surgery, hypoproteinemia, electrolyte imbalances, anemia, cerebrovascular disease, and heart failure. These key factors play a crucial role in determining the outcome of PLOS for elderly hip fracture patients and serve as strong predictive elements. Healthcare practitioners should pay special attention to these variables and prioritize early prevention strategies. Efficient allocation of medical resources to high-risk patients can enhance clinical effectiveness and reduce hospital stays, subsequently preventing complications associated with extended hospitalizations such as aspiration pneumonia, urinary tract infections, nosocomial infections, thrombosis, and intestinal obstruction. These measures are essential for improving the prognosis of elderly patients undergoing hip surgery. Among the ten critical variables, particular attention and monitoring should be given to patients with high-risk factors such as time from injury to admission, cerebrovascular disease, and heart failure. For other key variables like white blood cell count, D-dimer, hypoproteinemia, electrolyte imbalances, and anemia, clinicians should promptly intervene to prevent further deterioration. In patients without lower extremity vascular disease, health education, physical therapy, and pharmacological prevention should be emphasized; in those who have already developed the condition, immediate pharmacological treatment is necessary. Moreover, the study’s random forest algorithm was verified and compared using the Lasso regression model, lending significant clinical utility and practical application. This random forest predictive model furnishes clinicians with timely insights into the demands and risks associated with elderly hip fracture inpatients, enabling proactive interventions. It alleviates the risk factors associated with delayed hospital stays and enhances patient prognosis. In summary, this predictive model aids in optimizing bed turnover rates, reducing operational costs for healthcare facilities, minimizing unnecessary resource wastage, lowering treatment expenses for hospitalized patients, and enhancing postoperative outcomes.

Primarily, this study is inherently confined by its retrospective nature. Subsequently, the variation in hospitalization duration stems from disparities among different medical and caregiving systems. Thirdly, the study exclusively incorporates elderly hip fracture patients subjected to surgical interventions, revealing the discovery of individuals with a more extended temporal gap between data collection and non-surgical interventions. Regrettably, an analysis of this subgroup is absent. Despite the inclusion of an extensive array of variables, comprehensiveness remains elusive. R studio performs a data queue random assignment process, which is subject to uncertainty. The predictive model constructed in this study necessitates further validation through prospective research with a multicenter approach and a substantial sample size.

## 5 Conclusion

This study constructs a Random Forest predictive model, which is refined and validated through Lasso regression. This precise algorithm serves as a valuable tool for assessing personalized risk in elderly hip fracture patients. By applying the model, clinicians can develop tailored rehabilitation plans to improve patient prognosis. The model provides critical insights for clinical practitioners, aiding informed decision-making in the allocation of medical resources and the planning of healthcare interventions. This predictive model holds promise for providing clinicians with a theoretical basis for preventive interventions. Additionally, it facilitates the clinical translation of the model, enhancing its integration into practical medical use.

## Data availability statement

The original contributions presented in the study are included in the article/supplementary material, further inquiries can be directed to the corresponding authors.

## Ethics statement

The studies involving humans were approved by West China Hospital of Sichuan University. The studies were conducted in accordance with the local legislation and institutional requirements. Written informed consent for participation was not required from the participants or the participants’ legal guardians/next of kin in accordance with the national legislation and institutional requirements.

## Author contributions

HL: Writing – original draft. FX: Conceptualization, Methodology, Writing – original draft, Writing – review & editing. JJ: Investigation, Methodology, Project administration, Supervision, Writing – review & editing. ZC: Investigation, Methodology, Project administration, Writing – review & editing. ZX: Conceptualization, Supervision, Writing – review & editing. XD: Conceptualization, Data curation, Investigation, Project administration, Supervision, Writing – review & editing.
